# Dynamic Obstacle Avoidance with Enhanced Social Force Model and DWA Algorithm Using SparkLink

**DOI:** 10.3390/s25040992

**Published:** 2025-02-07

**Authors:** Hang Yi, Ruliang Lin, Hao Wang, Yifang Wang, Cunchao Ying, Dong Wang, Lihang Feng

**Affiliations:** 1 Beijing Aerospace Wanyuan Science and Technology Company Ltd., Beijing 100083, China; yihangcalt@163.com (H.Y.); linruliang2024@163.com (R.L.); omarwong@163.com (H.W.); wyf_785412@163.com (Y.W.); 2 School of Instrument Science and Engineering, Southeast University, Nanjing 210096, China; cunchaoying1994@163.com; 3 College of Electrical Engineering and Control Science, Nanjing Tech University, Nanjing 210096, China

**Keywords:** SparkLink, AGV, dynamic obstacle avoidance, social force model, dynamic window method

## Abstract

In the context of Industry 4.0, addressing the challenge of dynamic obstacle avoidance for Automated Guided Vehicles (AGVs) in complex industrial environments, this paper proposes an algorithm that integrates an enhanced social force model (SFM) and an improved dynamic window approach (DWA), leveraging SparkLink communication technology to enhance data transmission speed and reliability. The introduction of SparkLink technology significantly improves the environmental perception capabilities of AGVs, optimizing their dynamic obstacle-avoidance performance. Experimental results demonstrate that this method effectively increases the efficiency of AGVs in dynamic obstacle avoidance, offering significant practical value.

## 1. Introduction

**Figure 1:**
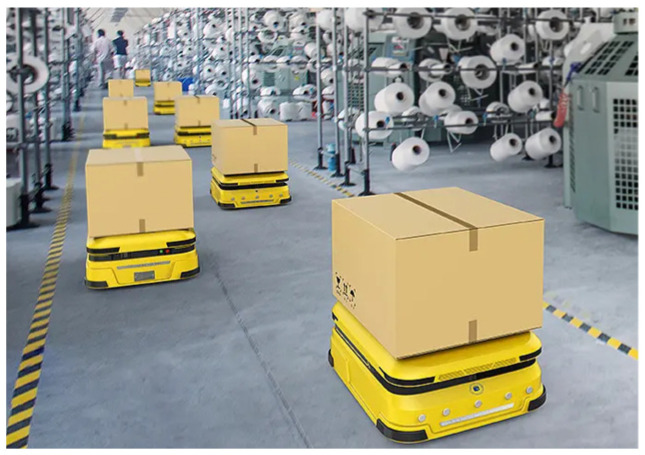
Photo of AGVs in operation.

In the era of Industry 4.0, the development of efficient material transport systems for intelligent production and large-part assembly is crucial as they can significantly enhance logistics management efficiency and the overall manufacturing process [1]. As shown in Figure 1, Automated Guided Vehicles (AGVs) navigate along pre-set paths within warehouses, automating the logistics and thereby boosting manufacturing efficiency [2].

However, one of the primary challenges faced by AGVs during material transport is dynamic obstacle avoidance, which directly impacts both path planning efficiency and the overall fluidity of logistics systems [3]. Effective obstacle-avoidance algorithms are essential to ensure that AGVs can respond promptly to obstacles, avoid collisions, and optimize energy consumption and operational efficiency. Therefore, the development and optimization of these algorithms are vital for improving the practical utility and competitiveness of AGVs, making them a key technology for realizing smart logistics and manufacturing [3].

In the field of dynamic obstacle avoidance, extensive research has been conducted, and various methods have been proposed to address this challenge. For example, Zhang [4] improved the Artificial Potential Field (APF) method by modifying the repulsive force range from a circular to an elliptical shape, adjusting it based on obstacle velocity. On the other hand, Model Predictive Control (MPC) can predict obstacle trajectories, and Olcay [5] combined it with Gaussian Process (GP) algorithms to estimate obstacle positions and generate avoidance trajectories. Zhu [6] integrated the APF method with MPC, where high-level MPC adjusted the APF avoidance path to generate a smooth trajectory, and low-level MPC followed this path for precise tracking.

In the case of dynamic window approach (DWA)-based avoidance, Hossain [7] enhanced the Gap-Following Method (GFM) and DWA by selecting more suitable gaps based on the size of the traversable space and the angle between the robot and its target. Additionally, the objective function of the DWA was improved to consider the benefits of passing through feasible gaps. Cheng [8] combined an improved A* algorithm with the DWA, modifying A* by checking the alignment of the current node, its parent node, and the grandparent node to reduce unnecessary turns and prune unnecessary expanded sub-nodes for global path planning, avoiding static obstacles, and then using the DWA to avoid dynamic obstacles during motion.

In terms of using deep learning for dynamic obstacle avoidance, He [9] combined the Deep Deterministic Policy Gradient (DDPG) with the APF method, where the APF was used to pre-plan a general avoidance direction. The DDPG network then adjusted the repulsive force parameters of the APF to autonomously learn and optimize the avoidance strategy. Similarly, Mulás-Tejeda [10] employed Long Short-Term Memory (LSTM) networks by placing multiple cameras around the robot to gather location data, along with the robot’s linear velocity, angular velocity, and distance to obstacles, which were all used for avoidance training of the LSTM network.

Other researchers have proposed alternative methods for dynamic obstacle avoidance. For instance, Yu [11] introduced a combined learning approach based on Sequential Neural Control Barrier Functions (SN-CBFs), where an SN-CBF model was designed to learn the time-series states of each dynamic obstacle, inferring safe control actions. By aggregating SN-CBF values, a unified value landscape was formed, from which optimal control actions for avoiding all obstacles were computed.

While current avoidance algorithms show excellent performance in their respective experimental settings, they generally overlook human-controlled dynamic obstacles that exhibit autonomous avoidance behaviors. Ignoring these behaviors can lead to suboptimal material transport, reducing overall production efficiency. Moreover, obstacle trajectory prediction is predominantly reliant on deep learning methods, which can produce unpredictable behaviors when faced with previously unseen scenarios during training. Additionally, factors such as the complexity of the working environment and network conditions (e.g., Wi-Fi latency and interference) can significantly affect the accuracy and timeliness of AGV data reception, further complicating real-time obstacle avoidance.

To address these challenges, this paper proposes an improved dynamic obstacle-avoidance algorithm that combines an enhanced Social Force Model (SFM) with the DWA and utilizes SparkLink communication technology. This algorithm incorporates human-controlled dynamic obstacles into the SFM, considering their avoidance behavior and integrating their predicted trajectories into the DWA objective function for more efficient avoidance. Furthermore, the SparkLink communication technology, known for its superior anti-interference capabilities and faster transmission speeds [12,13], enhances the algorithm’s performance in practical applications.

## 2. Environment Perception and Real-Time Data Transmission Based on SparkLink Technology

**Figure 2:**
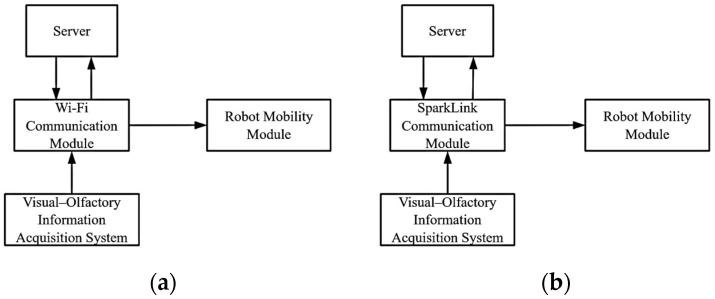
(**a**) The traditional robot communication architecture; (**b**) SparkLink robot communication architecture.

In the era of smart factories, decentralized control of AGV fleets offers enhanced flexibility and stability, enabling optimal resource utilization during varying production phases. Building on this control approach, this paper proposes an improvement to traditional communication methods by replacing the Wi-Fi module with a SparkLink module, as shown in Figure 2. AGVs need to continuously upload critical data such as position, speed, and remaining battery to the server to ensure smooth system operation. However, Wi-Fi limitations, including signal interference and latency, can lead to packet loss, increasing system unpredictability. In contrast, SparkLink technology offers obvious advantages in data transmission speed and reliability, enhancing the environmental perception capabilities of mobile robots.

The following sections describe the wireless communication architecture of SparkLink and its advantages in terms of environmental perception optimization in detail and compare and analyze the performance of the traditional wireless transmission module and the SparkLink module.

### 2.1. Environmental Perception Mechanism Based on SparkLink Technology

**Figure 3:**
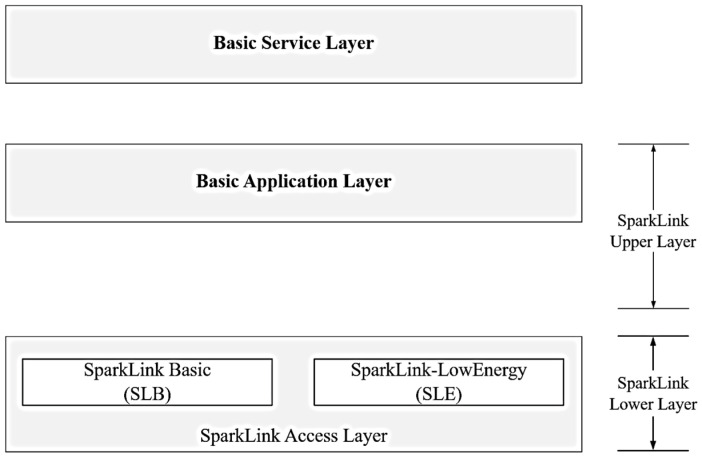
Wireless communication system architecture.

The wireless communication architecture [14] of SparkLink is shown in Figure 3, which provides two interfaces: SparkLink Low Energy (SLE) and SparkLink Basic (SLB). The SLE [12] is comparable to Bluetooth and is designed for low power consumption, low latency, and high reliability for applications such as wireless headsets and industrial data acquisition. The SLB [12], which is similar to Wi-Fi, is designed for smart devices and industrial machinery control and features low latency, high reliability, precise synchronization, and high concurrency.

In the realm of environmental perception systems, the ranging algorithm of SparkLink [15] has successfully overcome the precision challenges of traditional wireless technologies in complex environments. This advancement has enhanced the positioning accuracy from the meter to the decimeter level, which facilitates the exact determination of a mobile robot’s coordinates within a given scenario. When combined with the distance algorithms of a visual sensing system, this improvement allows for an even more precise identification of the current position of moving obstacles. Following this, an enhanced SFM algorithm can be employed to more accurately predict the position and velocity of moving obstacles at the next moment. Ultimately, the implementation of an improved DWA algorithm enables more precise dynamic obstacle avoidance, reducing the number of necessary stops and thereby increasing the efficiency of material transportation.

In the aspect of device communication, mobile robots can synchronize and transmit current battery level information to the SparkLink transmission module in a timely and accurate manner through the SLE interface. Subsequently, via the SLB interface, real-time data of the mobile robot’s speed, position, and remaining battery level are provided to the computing center.

### 2.2. Comparative Analysis of Conventional Wireless Transmission and SparkLink Modules

The relevant properties [12,16,17] required in industry for the conventional wireless transmission modules and SparkLink modules are shown in Table 1. SparkLink has significant advantages for industrial applications compared to Bluetooth and Wi-Fi 6. Its ultra-low latency of 20 µs (in SLB mode) outperforms both Wi-Fi 6 (1–5 ms) and Bluetooth (10 ms), making it ideal for real-time control in automation. SparkLink also offers decimeter-level positioning accuracy, which is more precise than Wi-Fi 6’s 5–10-m accuracy, benefiting tasks like asset tracking. Additionally, with a transmission speed of 900 Mbps (SLB mode) and power consumption of less than 2 mA, SparkLink provides a balanced solution for high-volume data transmission with low power usage, crucial for Industrial Internet of Things (IIoT) environments.

## 3. Adaptive Dynamic Obstacle-Avoidance Strategy Based on the SFM and the DWA

**Figure 4:**
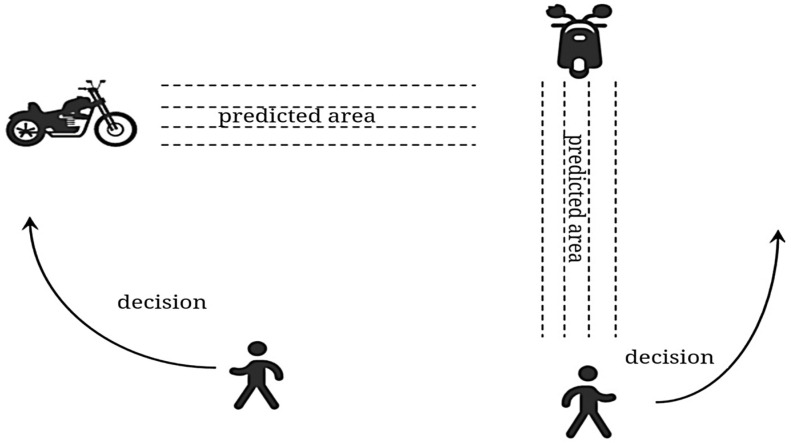
Walking strategy of a person when encountering obstacles.

As depicted in Figure 4, when individuals encounter other pedestrians or moving obstacles (such as motorcycles) during a walk, they first analyze the movement trends of these obstacles and then adjust their walking state based on their trajectory to preemptively avoid the positions that the obstacles are likely to pass through. Inspired by this observation, this paper proposes an obstacle-avoidance strategy that mimics human behavior when encountering dynamic obstacles: by predicting the behavior of moving obstacles and reacting in advance, the obstacle-avoidance path is optimized, thereby improving the efficiency of dynamic obstacle avoidance and maximizing productivity.

### 3.1. Pedestrian Trajectory Prediction

Pedestrian trajectory prediction is one of the key components of dynamic obstacle avoidance. Scholars worldwide have conducted extensive research in this field, proposing various pedestrian trajectory prediction models. Examples include models based on multi-environmental pedestrian behavior at crosswalks [18]; stochastic prediction models based on Dynamic Bayesian Networks (DBNs) [19]; prediction models based on Markov chains [20]; and the SFM, which analyzes the micro-dynamic characteristics of pedestrians [21].

Among these, the SFM, as a typical continuous pedestrian simulation model, has been widely applied in various fields, including traffic simulation, emergency evacuation simulation, and pedestrian trajectory prediction. The SFM transforms pedestrian movement behavior into mechanical interactions, using virtual force fields to simulate the interactions between pedestrians and between pedestrians and their environment. Specifically, pedestrians are influenced by the attraction to their goal, repulsion from others, and the repulsion from obstacles during their movement. Under the influence of the resultant force, they gradually adjust their motion state to ultimately reach their goal. The advantage of the SFM lies in its ability to accurately simulate pedestrian reactions and behaviors in dynamic environments.

To achieve more precise dynamic obstacle avoidance, this paper employs the SFM to predict the trajectories of pedestrians that a material-handling robot may encounter during its movement. The model assumes the following conditions: (1) pedestrians are solely influenced by external factors when moving or operating a vehicle, and there are no sudden avoidance behaviors triggered by internal causes (e.g., answering a phone); (2) the acceleration of pedestrians remains relatively constant within a given timeframe, without any abrupt changes in acceleration; (3) when pedestrians encounter obstacles, they actively take evasive actions. Building on this, the model’s obstacle-avoidance capabilities are further enhanced by incorporating the influence of moving obstacles on pedestrian avoidance behavior as a new parameter, thereby improving the efficiency of traditional obstacle-avoidance algorithms.

#### 3.1.1. The Traditional SFM

The SFM is based on the assumption that pedestrians are not only attracted to their goal but also experience repulsion from other pedestrians or obstacles. These forces collectively influence the pedestrian’s movement trajectory. In the traditional SFM, the motion of each pedestrian is influenced by three main forces:

- Self-motivation force: this force propels pedestrians toward their goal, representing their movement intentions;
- Interpersonal repulsion force: when two pedestrians come close to each other, a repulsive force is generated to prevent collisions;
- Boundary force: when pedestrians approach environmental boundaries or obstacles, a repulsive force is generated, forcing pedestrians to maintain a certain distance.

**Figure 5:**
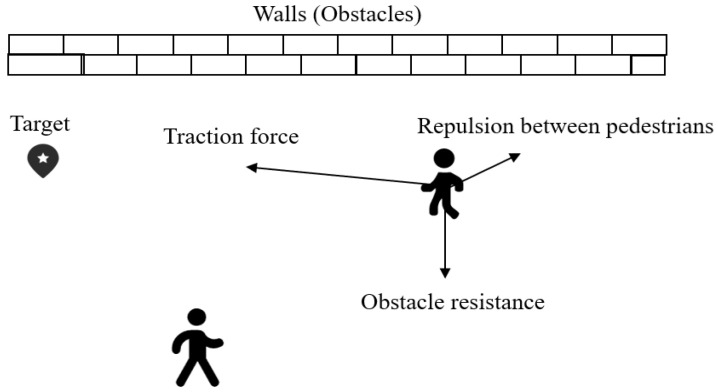
Schematic diagram of the original SFM.

The synthesis of these forces affects the pedestrian’s movement trajectory, thereby predicting their future position and direction of travel, as shown in Figure 5.

The kinematic equation of a pedestrian subjected to these three forces [21] is shown in Formula (1).

(1) $$F_{i}{=m}_{i}\frac{d_{\vec{v_{i}}}}{d_{t}}=\vec{F_{i}}+\sum_{j(j\neq i)}\vec{F_{\mathrm{ij}}}+\sum_{w}\vec{F_{\mathrm{iw}}}$$

where $\vec{F_{i}}$ is the self-driven force applied to the *i*th pedestrian, which describes the subjective expectation of the movement of that pedestrian toward the destination; $m_{i}$ is the mass of the *i*th pedestrian; $\sum_{j\left(j\neq i\right)}\vec{F_{\mathrm{ij}}}$ is the vector sum of the forces on pedestrian *i* for all pedestrians except pedestrian *i*; and $\sum_{w}\vec{F_{\mathrm{iw}}}$ is the vector sum of the forces acting on pedestrian *i* by the boundary or obstacle.

#### 3.1.2. The Improved SFM

This study refines the SFM by integrating mobile robots as obstacles and modeling pedestrians’ active avoidance behaviors as additional parameters. This enhancement facilitates a more detailed representation of pedestrian movement intentions, essential for adapting the model to complex scenarios involving pedestrian–environment and pedestrian–pedestrian interactions. The updated model (as shown in Formula (2)) offers a more precise characterization of pedestrian behavior in diverse settings.

(2) $$F_{i}{=m}_{i}\frac{d_{\vec{v_{i}}}}{d_{t}}=\vec{F_{i}}+\sum_{j(j\neq i)}\vec{F_{\mathrm{ij}}}+\sum_{w}\vec{F_{\mathrm{iw}}}+\vec{F_{\mathrm{ip}}}$$

where the term $\vec{F_{\mathrm{ip}}}$ represents the psychological driving force of the *i*th pedestrian. This characterizes the proactive avoidance behavior exhibited by pedestrians upon hearing obstacle-avoidance commands. In practical scenarios, the closer a pedestrian is to the robot, the more pronounced their active avoidance behavior becomes.

**Figure 6:**
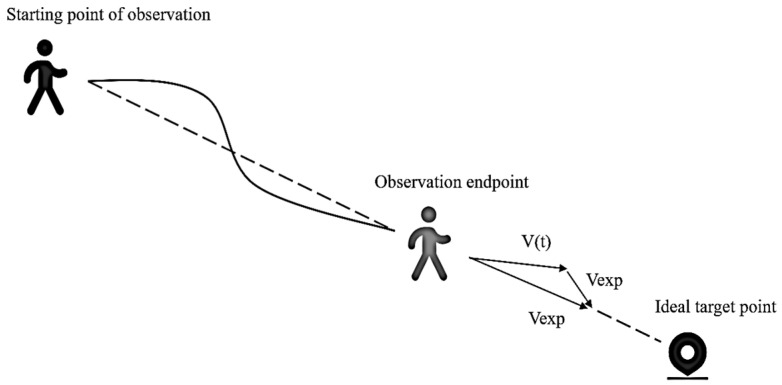
Determination of the ideal direction of the pedestrian.

A pedestrian is motivated to travel at a certain speed along the shortest path if undisturbed. In addition, if the pedestrian is disturbed by other pedestrians or the surrounding environment, their walking direction will change, and then the pedestrian will gradually return to the original walking direction under the action of the driving force. The expression for the driving force $\vec{F_{i}}$ [22] is shown in Formula (3):

(3) $$\vec{F_{i}}{=m}_{i}\frac{v_{\exp}\cdot \vec{e_{i}}{\text{-}v}_{i}\left(t\right)}{\tau }$$

where $v_{exp}$ is the desired velocity scalar of pedestrian *i*; $\vec{e_{I}}$ is the unit direction vector representing the desired movement direction; $v_{i}\left(t\right)$ is the actual velocity of pedestrian i at moment *t*; and τ is the reaction time of the pedestrian to the change in acceleration, which is the relaxation time when pedestrian *i* adjusts the current speed to the desired speed. In order to determine the ideal walking direction of the pedestrian, the pedestrian in front of the mobile robot can be observed using the various on-board sensors of the mobile robot, and then the line connecting the starting and ending points of the pedestrian walking during this observation time Δ*T* can be used as this direction, as shown in Figure 6.

$\sum_{j\left(j\neq i\right)}\vec{F_{\mathrm{ij}}}$ in Formula (2) is the vector sum of the forces on pedestrian *i* for all pedestrians except pedestrian *i*. This force is used to describe the current tendency of a pedestrian to avoid walking with another person when they are approaching. The expression of $\vec{F_{\mathrm{ij}}}$ [22] is shown in Formula (4):

(4) $$\vec{F_{\mathrm{ij}}}{=A}_{i}\exp(\frac{r_{i}{+r}_{j}{\text{-}d}_{\mathrm{ij}}}{B_{i}})\vec{e_{\mathrm{ij}}}$$

where $A_{i}$ and $B_{i}$ are force strength and action range constants, respectively; $r_{i}$, $r_{j}$ the radius of pedestrian *i* and *j*, respectively. $d_{ij}$ represents the distance between the centroids of pedestrians *i* and *j*. Additionally, $\vec{e_{\mathrm{ij}}}$ is a unit vector indicating the direction of the force exerted from pedestrian *j* to pedestrian *i*.

The $\sum_{w}\vec{F_{\mathrm{iw}}}$ [22] in Formula (2) is the vector sum of the forces acting on pedestrian *i* by the boundary or obstacle. Similar to the pedestrian-to-pedestrian force, this force describes the tendency of pedestrians to exhibit a certain distance from a fixed obstacle, such as a control boundary or a post, when they are in close proximity to it during walking. Since the psychological driving force represents the intention of pedestrians to avoid mobile robots, it is necessary to refine the traditional SFM by eliminating the complex maneuvering associated with pedestrians navigating around obstacles. This modification not only simplifies the model but also accelerates the computational speed of the program. The revised formula is shown in Formula (5):

(5) $$\vec{F_{\mathrm{iw}}}{=A}_{w}\exp(\frac{r_{i}{\text{-}d}_{\mathrm{iw}}}{B_{w}})\vec{e_{\mathrm{iw}}}$$

where $A_{w}$ and $B_{w}$ are the force strength and action range constants, respectively; $r_{i}$ is the radius of pedestrian *i*; $\vec{e_{\mathrm{iw}}}$ is a unit vector indicating the direction of the force pointing from the boundary or obstacle to pedestrian *i*, taken to be perpendicular to the boundary or obstacle direction; and $d_{\mathrm{iw}}$ is the distance between the pedestrian and the boundary.

Since the introduction of the SFM by Dirk Helbing and Peter Molnar, many scholars have proposed a series of variants of the social force model based on different application scenarios [23,24,25]. In order to apply the SFM to the scenario studied in this paper, an additional psychological driver is introduced on top of the above three forces. If a material-transport robot encounters a pedestrian ahead while moving, it typically issues a voice alert to prompt the pedestrian to move out of the way. Consequently, the psychological driving force can be employed in practical scenarios to account for the pedestrian’s evasive maneuvers not considered in Formula (5), particularly when pedestrians are required to navigate around corners. This driving force is represented by Formula (6):

(6) $$\vec{F_{\mathrm{ip}}}{=A}_{p}\exp(\frac{r_{i}{\text{-}d}_{\mathrm{ip}}}{B_{p}})\vec{e_{\mathrm{ip}}}$$

where $A_{p}$ and $B_{p}$ are the intensity and range coefficients of the pedestrian mental drive, respectively; $r_{i}$ is the distance between the pedestrian and the robot; $d_{ip}$ is the direction of the pedestrian velocity change; and $\overset{⃑}{e_{\mathrm{ip}}}$ is the unit vector indicating the direction of the pedestrian velocity change. After calculating the combined force applied to a single pedestrian, the combined force can be directly used as acceleration without considering the mass of the pedestrian, and then its position can be recursively projected according to Formula (7) based on the time step for the purpose of predicting the trajectory of the pedestrian in the future period. In Formula (7), $p_{t}$ and $p_{t+1}$ are the positions of pedestrians at time *t* and *t* + 1; $v_{t}$ and $v_{t+1}$ are the speed of pedestrians at time *t* and *t* + 1; and $a_{t}$ is the acceleration at time *t*.

(7) $$\left\{\begin{array}{c} p_{t+1}{=p}_{t}{+v}_{t}{\Delta t+0.5a}_{t}{\Delta t}^{2}\\ v_{t+1}{=v}_{t}{+a}_{t}\Delta t \end{array}\right.$$

### 3.2. Adaptive Obstacle-Avoidance Strategy

The proposed adaptive obstacle-avoidance strategy incorporates an enhanced SFM with the DWA algorithm, adjusting dynamically to environmental settings. In narrow spaces, the AGV maintains safe distances using the SFM to preempt collisions. In spacious scenarios, the AGV integrates the improved DWA algorithm with trajectory predictions for optimized obstacle avoidance, ensuring efficient and smooth path navigation.

#### 3.2.1. Narrow-Space Obstacle-Avoidance Strategy

**Figure 7:**
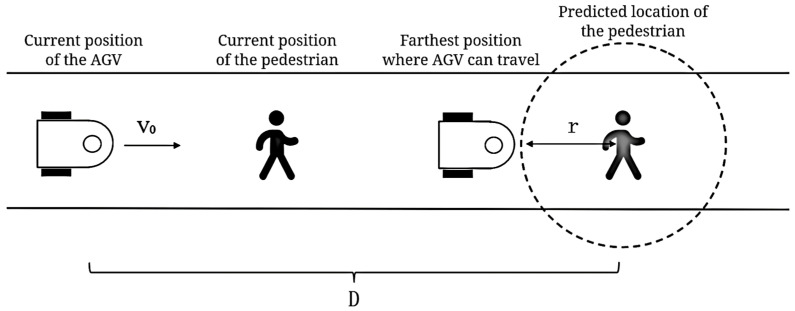
Obstacle avoidance of material-transport robot in narrow corridor.

The narrow-space obstacle-avoidance strategy of the AGV is shown in Figure 7.

When the AGV encounters a pedestrian in the narrow corridor of the factory, the traveler’s walking position after a period of time can first be predicted by the SFM. After that, a danger radius r is determined, and the farthest position of the AGV walking can be obtained, as shown in Figure 7. If the current velocity of the AGV is $v_{0}$, the distance from the current position to the farthest position that can be walked to is D. Assuming that the mobile robot performs uniform variable speed linear motion, the acceleration of the AGV can be derived from the kinematic equation shown in Formula (8).

(8) $$a=\frac{2\cdot (D\text{−}r)}{{∆T}^{2}}-\frac{2\nu_{0}}{∆T}$$

where ∆T is the predicted duration. By iterating according to this formula, the process is streamlined through continuous adjustments to the mobile robot’s speed, which are based on the calculated acceleration. This ensures that the robot maintains a safe distance from the pedestrian while navigating through the environment. Specifically, the maximum distance *D* that the robot can travel within this predicted duration can be determined using the following Formula (9):

(9) $$D=\sqrt{{\left(x_{obn}-x_{current}\right)}^{2}+{\left(y_{obn}-y_{current}\right)}^{2}}$$

where $\left(x_{current},y_{current}\right)$ denotes the current coordinates of the mobile robot; and $\left(x_{obn},y_{obn}\right)$ signifies the anticipated position of the pedestrian after the predicted interval. Additionally, the danger radius *r*—which is the minimum distance the robot should maintain from the pedestrian to avoid collision—is a preset value.

#### 3.2.2. Spacious-Environment Obstacle-Avoidance Strategy

In expansive settings, AGVs employ an enhanced DWA algorithm for dynamic obstacle avoidance, which is an improvement upon the algorithm proposed by Fox et al. [26] based on the Curvature Velocity Method (CVM) [27]. This approach converts positional constraints to velocity constraints, accounting for mechanical properties and environmental obstacles. Integrated with an improved SFM, AGVs predict obstacle trajectories and refine avoidance paths, ensuring swift responses to dynamic obstacles and efficient navigation.

- The traditional DWA algorithm

**Figure 8:**
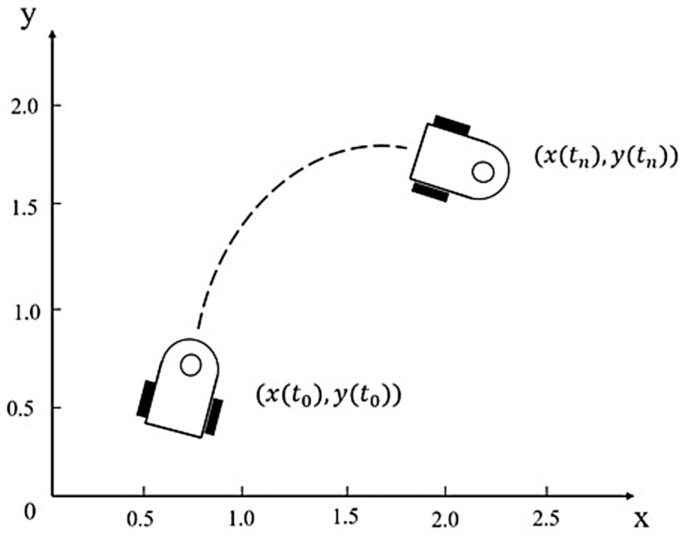
Kinematic model of the mobile robot.

In the conventional DWA algorithm, the kinematic model of the robot is first established as shown in Figure 8 since the trajectory prediction is performed for all states within the window of the mobile robot.

In the figure, $v\left(t\right)$ and $\omega \left(t\right)$ are the linear and angular velocities of the mobile robot, respectively. Since the sampling period is extremely short, the curved motion trajectory of the robot can be approximated as a straight line. If the coordinates of the current robot are $x\left(t\right)$, $y\left(t\right)$, and $\theta \left(t\right)$, the position at the next sampling moment can be expressed by Formula (10):

(10) $$\left\{\begin{array}{c} x\left(t+1\right)=x\left(t\right)+v(t)\Delta tcos(\theta (t))\\ y\left(t+1\right)=y\left(t\right)+v(t)\Delta tsin(\theta (t))\\ \theta \left(t+1\right)=x\left(t\right)+\omega (t)\Delta t \end{array}\right.$$

According to the kinematic model of Formula (8), the trajectory of the robot after a predicted period of time can be obtained. The speed of the robot at the next moment is limited to a window considering the constraints of the robot itself and its surroundings. The window considers three factors. First, the maximum speed limit of the robot, as shown in Formula (11):

(11) $$V_{1}=\left\{\left(v,\omega \right)\right|0\leq v{\leq v}_{\max},\;0\leq \omega {\leq \omega }_{\max}\text{\}}$$

Second, considering the constraints of the driving force of the mobile robot, there is a maximum acceleration and deceleration limit. If the current linear velocity is $v_{0}$ and the angular velocity is $w_{0}$, the velocity limit at the next moment is as in Formula (12):

(12) $$V_{2}=\left\{\left(v,\omega \right)\right|v_{0}\text{-}a\Delta t\leq v\leq v_{0}+a\Delta t,\;\omega_{0}{\text{-}a}_{w}\Delta t\leq \omega \leq \omega_{0}{+a}_{\omega }\Delta t\text{\}}$$

Third, considering the safety of the robot, in order to be able to stop before hitting an obstacle, the speed limit of the robot with the maximum deceleration is shown in Formula (13):

(13) $$V_{3}=\left\{\left(v,\omega \right)\right|\;v\leq \sqrt{2\cdot \mathrm{dist}\left(v,\omega \right)\cdot v},\;\omega \leq \sqrt{2\cdot \mathrm{dist}\left(v,\omega \right)\cdot \omega }\text{\}}$$

where $dist\left(v,\omega \right)$ in Formula (11) is the distance between the endpoint of the track corresponding to speed $\left(v,\omega \right)$ and the obstacle. To sum up, the final speed window of the mobile robot is the intersection of three sets, as shown in Formula (14):

(14) $${V=V}_{1}{\text{∩}V}_{2}{\text{∩}V}_{3}$$

**Figure 9:**
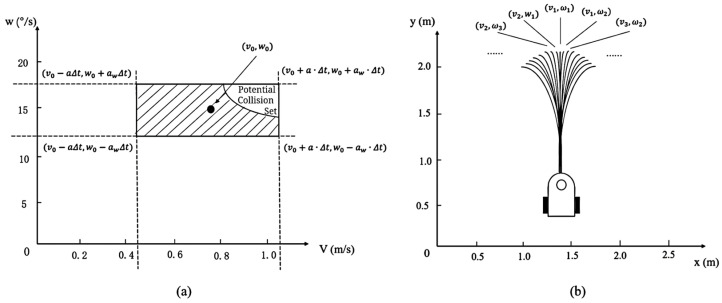
Kinematic model of a typical mobile robot. (**a**) The mobile robot determines the final window; (**b**) The multiple trajectories sampled by the robot within the dynamic window.

The final window *V* and the sampling trajectory formed by *V* are shown in Figure 9.

**Figure 10:**
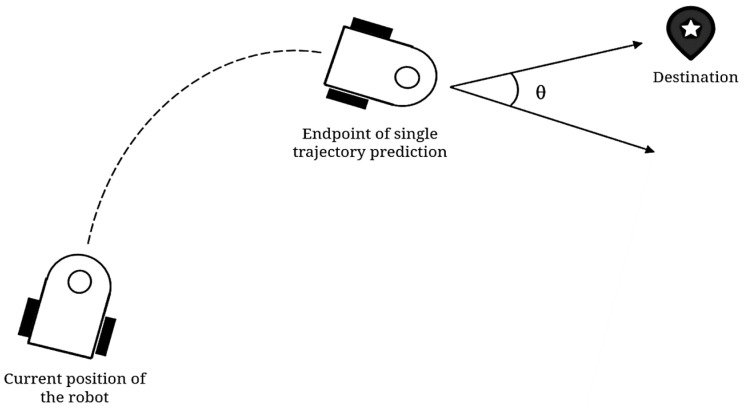
Schematic diagram of heading evaluation items.

Among the obtained trajectories, the trajectories corresponding to each set of velocities are feasible. In order to obtain the optimal trajectory, each trajectory needs to be evaluated and the one with the highest score is selected as the optimal solution and executed. The evaluation function is shown in Formula (15):

(15) $$G\left(v,w\right)=\sigma (\alpha \cdot \mathrm{heading}\left(v,w\right)+\beta \cdot \mathrm{dist}\left(v,w\right)+\gamma \cdot \mathrm{velocity}(v,w))$$

where the function term $heading\left(v,w\right)$ in Formula (13) is the orientation angle, which is used to evaluate the angular difference between the direction of the end of the redirected trajectory and the target point, as shown in Figure 10. This is expressed by the following Formula (16):

(16) heading=180°- θ

The function $dist\left(v,\omega \right)$ is used to evaluate the distance between the end of the robot’s current trajectory and the nearest obstacle. To prevent this term from being over-evaluated, it needs to be set to a constant for scoring paths without obstacles; therefore, the function term $velocity\left(v,\omega \right)$ is used to evaluate the velocity corresponding to this trajectory, and this evaluation allows the mobile robot to approach the target point at a faster speed.

Since the above three evaluation terms have different meanings and units, they need to be normalized after calculating the three evaluation values with Formula (17):

(17) $$\left\{\begin{array}{c} {\mathrm{heading}}_{\mathrm{normal}}=\frac{\mathrm{heading}(i)}{\sum_{i=1}^{n}\mathrm{heading}(i)}\\ {\mathrm{dist}}_{\mathrm{normal}}=\frac{\mathrm{dist}(i)}{\sum_{i=1}^{n}\mathrm{dist}(i)}\\ {\mathrm{velocity}}_{\mathrm{normal}}=\frac{\mathrm{velocity}(i)}{\sum_{i=1}^{n}\mathrm{velocity}(i)} \end{array}\right.$$

The calculated three evaluation values are brought into Formula (15) to obtain the rating of the current state, and then the highest rated set $\left(v,\omega \right)$ is selected from all the states in the window as the state for the next moment, which is the overall process of the traditional dynamic window.

- The improved DWA algorithm

**Figure 11:**
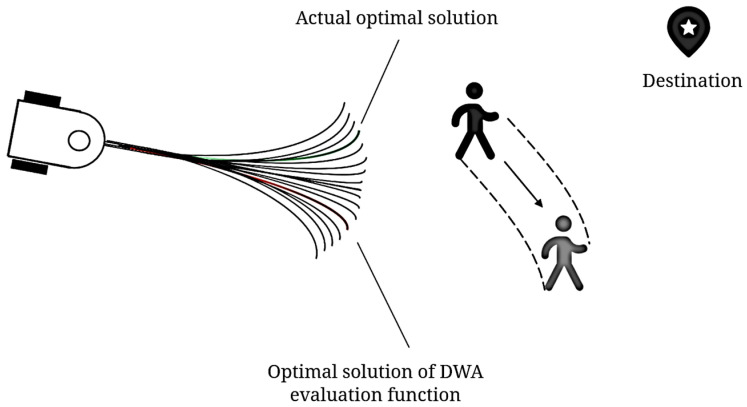
Schematic diagram of conventional DWA facing dynamic obstacle.

The DWA obstacle-avoidance algorithm, widely used in mobile robotics, is significantly less efficient when dealing with dynamic obstacles. As shown in Figure 11, the conventional DWA predicts states and evaluates the distance between predicted trajectories and the pedestrian’s current position, which is optimal for stationary pedestrians. However, with moving pedestrians, this approach can lead to suboptimal solutions due to local optimization, where the robot’s movement aligns with the pedestrian’s motion, neglecting global optimality.

**Figure 12:**
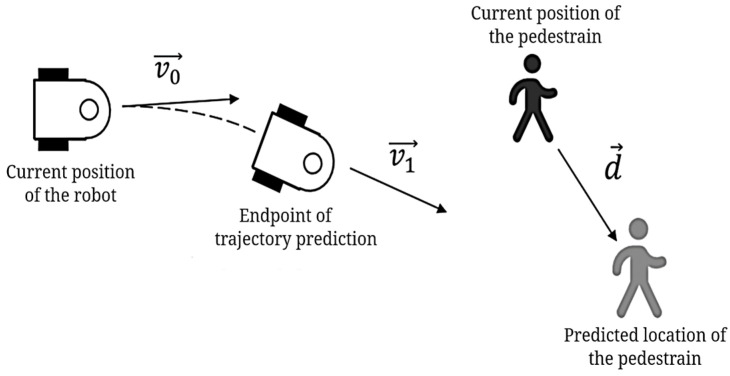
Schematic diagram of the evaluation term: *predict*(*v*, *ω*).

To enhance obstacle anticipation and improve safety in tasks such as drug delivery, this paper introduces a novel evaluation term, $predict\left(v,\omega \right)$, to the DWA algorithm. This term integrates the robot’s trajectory endpoint and the pedestrian’s predicted location from the SFM, as demonstrated in Figure 12. By incorporating $predict\left(v,\omega \right)$, the algorithm can better predict the movement of dynamic obstacles, optimize avoidance paths, and ensure efficient navigation.

In Figure 12, $\vec{v_{0}}$ is the current velocity vector of the mobile robot; $\vec{v_{1}}$ is the velocity vector of the robot at the end of the trajectory prediction; and $\vec{d}$ is the vector of the pedestrian’s current position pointing to the predicted position. Therefore, $predict\left(v,\omega \right)$ can be expressed by Formula (18):

(18) $$\mathrm{predict}\left(v,\omega \right)=\left\{\begin{array}{c} ∠\left(\vec{v_{1}},\;\vec{d}\right)∠\left(\vec{v_{0}},\;\vec{d}\right)<90{}^{\circ}\\ 180{}^{\circ}\text{-}∠\left(\vec{v_{1}},\;\vec{d}\right)∠\left(\vec{v_{0}},\;\vec{d}\right)\geq 90{}^{\circ} \end{array}\right.$$

**Figure 13:**
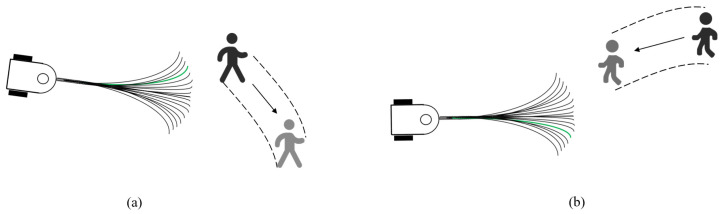
Preferred trajectory of the mobile robot in two cases.(**a**) the angle between the robot’s current velocity direction and the pedestrian’s movement direction is less than 90°; (**b**) the angle between the robot’s current velocity direction and the pedestrian’s movement direction is more than 90°.

When the angle between the robot’s current velocity direction and the pedestrian’s movement direction is less than 90°, it indicates that the robot is moving in roughly the same direction as the pedestrian. In this case, the robot should prioritize trajectories that increase the distance between itself and the pedestrian to avoid trailing too closely, as shown in Figure 13a. Conversely, when the angle between the robot’s velocity direction and the pedestrian’s direction is greater than 90°, the robot is moving in the opposite direction from the pedestrian. In this scenario, the robot should select trajectories that reduce the angle and prevent a head-on collision by steering away from the pedestrian’s path, as shown in Figure 13b.

Likewise, this scoring term needs to be normalized after it is calculated, as shown in Formula (19):

(19) $${predict}_{normal}=\frac{predict\left(i\right)}{\sum_{i=1}^{n}predict\left(i\right)}$$

Finally, the robot’s overall evaluation function is updated to include the prediction term, as shown in Formula (20):

(20) $$G\left(v,w\right)=\sigma (\alpha \cdot \mathrm{heading}\left(v,w\right)+\beta \cdot \mathrm{dist}\left(v,w\right)+\gamma \cdot \mathrm{velocity}\left(v,w\right)+\varepsilon \cdot \mathrm{predict}\left(v,w\right))$$

In the evaluation Function (20), the robot considers multiple factors: heading toward the goal, distance from obstacles, speed, and the predicted movement of pedestrians. The optimal path is selected by choosing the trajectory that scores the highest based on this comprehensive evaluation, ensuring safe and efficient navigation in dynamic environments.

## 4. Simulation Verification and Result Analysis

### 4.1. Simulation Setup

To assess the efficacy of the obstacle-avoidance algorithm presented in this paper, comparative tests were conducted in both narrow aisles and large warehouses under identical pedestrian scenarios. The experiments primarily involved parameters from three areas—the mobile robot’s specifications, the DWA algorithm settings, and the SFM parameters—as detailed in Table 2, Table 3 and Table 4.

In these simulation experiments, the AGV’s kinematic data—position, velocity, and angular velocity—is gathered through an array of sensors. The AGV’s position is ascertained using simulated GPS and LiDAR sensors, which provide real-time coordinate calculations. Velocity is determined via IMUs or wheel encoders: IMUs supply data on linear and angular velocities, while wheel encoders calculate the AGV’s speed based on wheel rotation. In addition, angular velocity is predominantly sourced from IMUs. The control program regularly retrieves sensor data through the simulation platform’s interfaces to refresh the AGV’s motion status.

To simulate real-world communication conditions, data transmitted from the AGV to the control system pass through a network delay module. The delay configuration of this module is tailored according to the communication technology used. According to Table 1, the latency of SparkLink communication technology is set at 20 nanoseconds, while the latency of Wi-Fi communication is set between 1 and 5 milliseconds. This approach allows for a more accurate replication of the AGV’s performance under different communication technologies and provides a comparative analysis of data transmission between SparkLink and traditional Wi-Fi, ensuring that the research findings have practical significance.

### 4.2. Narrow-Corridor Simulation Results

**Figure 14:**
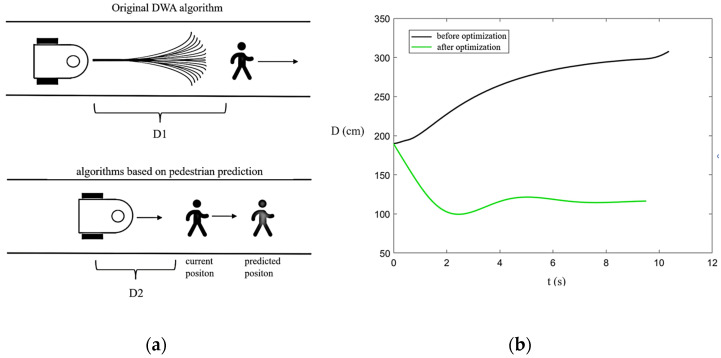
Comparison of the effect of obstacle avoidance.(**a**) Comparison of Original and Improved DWA Algorithms in Narrow Scenarios; (**b**) Simulation Results for Narrow Scenarios.

As shown in Figure 14a, a comparison of the actual performance between the original DWA algorithm with Wi-Fi and the AGV’s narrow-corridor obstacle-avoidance model under the same experimental conditions is presented.

Figure 14b presents the distance–time plot showing the relative distance between the robot and pedestrians during the experiment. In this scenario, both the robot and the pedestrian move in the same direction. The robot’s initial speed is 0.7 m/s, while the pedestrian moves at 0.8 m/s. The initial distance between the robot and the pedestrian is 1.9 m, with a hazard radius of 1.5 m defined in Formula (8).

The black curve in Figure 14b represents the performance of the original DWA algorithm with Wi-Fi. Given the algorithm’s lack of consideration for the intentions of pedestrians, by the termination of the experimental timeframe, the mobile robot’s trajectory resulted in a sustained separation of approximately 3 m from the pedestrian. In contrast, the optimized algorithm predicts the pedestrian’s future movement and adjusts the robot’s path accordingly, reducing the distance between the robot and the pedestrian to approximately 1.2 m after stabilization. This improvement is attributed to the algorithm’s ability to determine that the pedestrian will continue walking forward, allowing the robot to maintain a closer yet safe proximity.

### 4.3. Wide-Area Simulation Results

#### 4.3.1. Comparison of Single-Pedestrian Obstacle-Avoidance Effect

**Figure 15:**
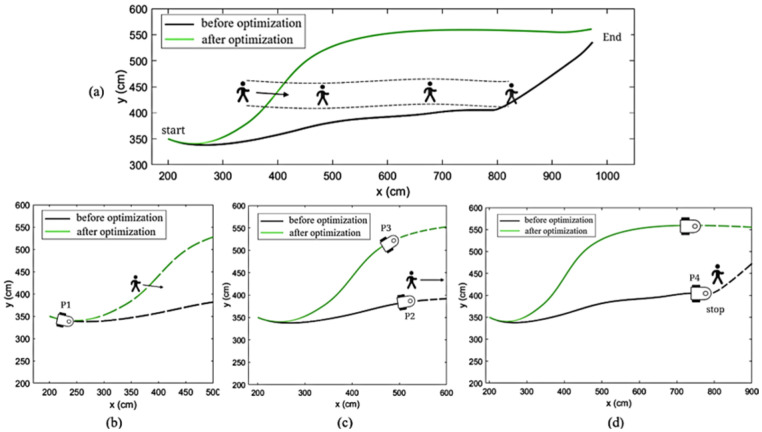
Comparison of the effect of single-pedestrian obstacle avoidance. (**a**) Overall obstacle-avoidance trajectories of the original DWA algorithm and the optimized DWA algorithm in the presence of a single pedestrian; (**b**) Initial phase of the simulation; (**c**) Mid-phase of the simulation; (**d**) The original DWA algorithm encounters a pedestrian, and the experiment stops.

This simulation examines the effectiveness of the improved algorithm in avoiding a single pedestrian in a wide hall environment. The pedestrian moves from point (250, 430) to (800, 430), as depicted in Figure 15. The dashed line in Figure 15a represents the pedestrian’s path, while the black curve below indicates the real-time path planned by the original DWA algorithm with Wi-Fi. In contrast, the green curve above shows the path planned by the wide-area obstacle-avoidance model of the AGV.

As illustrated in Figure 15b, the mobile robot approaches the pedestrian at point P1 and enters the pedestrian’s influence range. As the pedestrian continues to move, the robot, following the original DWA with Wi-Fi evaluation function, selects an optimal trajectory. However, since the original algorithm with Wi-Fi cannot predict the pedestrian’s next position, the robot continues to avoid the pedestrian’s walking path after point P2, as shown in Figure 15c. Eventually, the robot halts at position P4 (where the window size is 0) and does not resume its movement toward the target point until the pedestrian exits its influence range. During the entire process, the DWA evaluation function was executed 220 times, with a total running time of 11.35 s.

In contrast, the improved algorithm anticipates the pedestrian’s future trajectory, allowing the robot to adjust its path earlier to avoid the predicted direction of pedestrian movement. Once the robot passes point P3 in Figure 15c, it is no longer affected by the pedestrian, which significantly reduces the interaction time and enhances the safety of the robot’s obstacle avoidance. The improved algorithm completed the obstacle-avoidance process in 160 iterations, with a total running time of 8.79 s. The comparison of the two algorithms is provided in Table 5.

#### 4.3.2. Multiple-Pedestrian Obstacle-Avoidance Simulation

**Figure 16:**
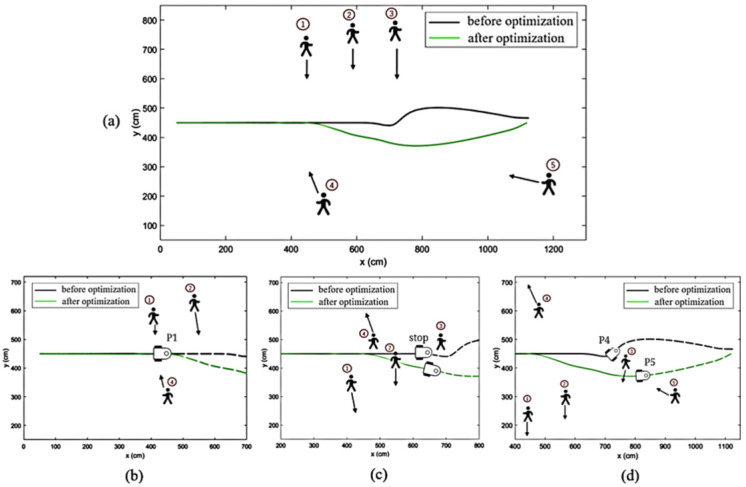
Comparison of the effect of multi-pedestrian obstacle avoidance.(**a**) Overall obstacle-avoidance trajectories of the original DWA algorithm and the optimized DWA algorithm in the presence of a single pedestrian; (**b**) Initial phase of the simulation; (**c**) Mid-phase of the simulation; the original DWA algorithm encounters a pedestrian and stops to wait.; (**d**) Late phase of the simulation.

This simulation evaluates the obstacle-avoidance performance of the mobile robot when encountering multiple pedestrians in a spacious warehouse setting. As depicted in Figure 16, five pedestrians are involved in the simulation, with their movement directions indicated by black arrows. Pedestrians 1 and 4 decelerate when they encounter the robot, exhibiting avoidance behavior, while the remaining pedestrians continue walking without initiating avoidance.

As shown in Figure 16b, the original DWA algorithm with Wi-Fi approaches pedestrian 2 at point P1 after successfully passing pedestrians 1 and 4. However, due to the algorithm’s inability to predict pedestrian movement, the robot’s trajectory is not adjusted in time, leading to a conflict with pedestrian 3 at point P2, as illustrated in Figure 16c. The robot then slows down and comes to a stop before bypassing pedestrian 3. Throughout the obstacle-avoidance process, the original DWA algorithm with Wi-Fi executed 311 evaluations, with a total avoidance time of 15.5 s.

In contrast, the optimized DWA algorithm with SparkLink, as shown in Figure 16a, predicts pedestrian 2’s downward movement at point P1, allowing the robot to proactively avoid pedestrian 2. This early prediction provides sufficient time for the robot to bypass pedestrian 3 at point P3 without stopping, as seen in Figure 16b. The robot later encounters pedestrian 5 at point P5 in Figure 16d and successfully maneuvers upward to reach the target destination. The improved DWA algorithm executed 225 evaluations during the entire process, reducing the total obstacle-avoidance time to 11.2 s. The comparison between the two algorithms is summarized in Table 6.

This comparison highlights the enhanced efficiency and reduced execution time of the optimized DWA algorithm, particularly in multi-pedestrian scenarios. The ability of the improved algorithm to predict pedestrian movement leads to more proactive and safer obstacle-avoidance strategies.

## 5. Conclusions

This article introduces a method for dynamic obstacle avoidance using SparkLink communication, which combines an enhanced SFM and an improved DWA. This approach aims to enhance the capabilities of material-handling robots in industrial mass-production environments, reducing the risk of employee injuries or reduced production efficiency due to suboptimal obstacle avoidance caused by poor network conditions. By leveraging SparkLink positioning technology to obtain accurate information required for the improved SFM, mobile robots are able to score each subsequent state based on the evaluation function in the improved DWA algorithm, with the highest-scoring state dictating the motion trajectory. Experimental results indicate that the enhanced algorithm outperforms the original DWA algorithm equipped with Wi-Fi in terms of both obstacle avoidance and transportation efficiency, as evidenced by its exceptional performance in the simulation environments constructed for this purpose and validated through representative single- and multi-pedestrian scenarios.

The primary focus of this paper is to improve the original obstacle-avoidance algorithm for mobile robots based on the prediction of pedestrian walking intentions using SparkLink technology. However, in real-world scenarios, factors such as age, gender, and individual differences among pedestrians can affect the predictive accuracy of the SFM [28,29,30]. Additionally, the simulation of SparkLink in this experiment mainly remains at the theoretical data stage, without specific testing under various network conditions. Although delay parameters were set to simulate the low latency and high stability of SparkLink based on the characteristics of the communication technology, a comprehensive experimental verification of SparkLink’s performance under different network conditions was not conducted. Future work will involve more extensive testing of SparkLink communication technology under diverse network environments to assess its performance and reliability in practical applications.

## Figures and Tables

**Table 1:** Performance comparison of traditional wireless transmission and SparkLink modules.

	Transmission Speed	Latency	Positioning Accuracy	Power Consumption
Bluetooth	1~2 Mbps	10 ms	Centimeter-level	1~2 mA
Wi-Fi 6	$9.6\;Gbps\left(MAX\right)$	1~5 ms	5~10 m	>2mA
SparkLink	SLE:12 Mbps SLB:900 Mbps	SLE:250 μs SLB:20 μs	Decimeter-level	<2mA

**Table 2:** Parameters of the mobile robot.

Name	Value	Unit
Minimum Line Speed: V_min_	0	m/s
Maximum Linear Speed: V_max_	1.2	m/s
Maximum Linear Acceleration: a_max_	1	m/s^2^
Maximum Angular Velocity: ω_max_	60	deg/s
Minimum Angular Velocity: ω_min_	−60	deg/s
Maximum Angular Acceleration: a_ω_	60	deg/s^2^

**Table 3:** Parameters of the DWA algorithm.

Name	Value	Unit	Related Formulas
Resolution of Time: Δt	0.05	s	(10)
Resolution of Linear Speed: Δv	0.01	m/s	(14)
Resolution of Angular Velocity: Δω	1	deg/s	(14)
Time of Trajectory Prediction: test	2	s	(8)
Maximum Obstacle Distance Threshold: dist	0.4	m	—
Weights for Azimuth Evaluation: α	0.1	—	(15) and (20)
Weights for Obstacle Distance Evaluation: β	0.2	—	(15) and (20)
Weights of Robot Speed Evaluation: γ	0.1	—	(15) and (20)
Weights for Predicting Pedestrian Evaluations	0.1	—	(20)

**Table 4:** Parameters of SFM.

Name	Value	Unit	Related Formulas
Strength of Pedestrian Interaction Forces: Ai	0.8	—	(4)
Constant of Pedestrian Interaction Range: Bi	1.85	—	(4)
Strength of Obstacle Force: Aw	0.4	—	(5)
Obstacle Force Range Constant: Bw	0.9	—	(5)
Strength of Psychological Forces on Pedestrians: Ap	0.5	—	(6)
Pedestrian Psychological Force Range Constant: Bp	2.0	—	(6)

**Table 5:** Comparison of obstacle-avoidance results of experiment Section 4.3.1.

Algorithm	Number of Iterations	Total Time (s)	Total Distance (m)
Original DWA algorithm with Wi-Fi	220	11.35	12.1
Improved DWA algorithm with SparkLink	160	8.79	10.73

**Table 6:** Comparison of multi-pedestrian obstacle-avoidance results in the lobby.

Algorithm	Number of Iterations	Total Time (s)	Total Distance (m)
Original DWA algorithm with Wi-Fi	311	15.5	12.3
Improved DWA algorithm with SparkLink	225	11.2	12.1

## Data Availability

The data presented in this study are available on request from the corresponding author due to privacy and ethical restrictions.
